# Mechanisms of biodiversity between *Campylobacter* sequence types in a flock of broiler–breeder chickens

**DOI:** 10.1002/ece3.8651

**Published:** 2022-03-06

**Authors:** Thomas Rawson, Frances M. Colles, J. Christopher D. Terry, Michael B. Bonsall

**Affiliations:** ^1^ 6396 Department of Zoology, Mathematical Ecology Research Group University of Oxford Oxford UK; ^2^ 6396 Department of Zoology Peter Medawar Building for Pathogen Research University of Oxford Oxford UK; ^3^ 6396 NIHR Health Protection Research Unit in Gastrointestinal Infections University of Oxford Oxford UK; ^4^ 4617 School of Biological and Behavioural Sciences Queen Mary University of London London UK

**Keywords:** biodiversity, broiler, *Campylobacter*, microbial competition, sequence type

## Abstract

Commercial poultry flocks frequently harbor the dangerous bacterial pathogen *Campylobacter*. As exclusion efforts frequently fail, there is interest in potential ecologically informed solutions. A long‐term study of *Campylobacter* sequence types was used to investigate the competitive framework of the *Campylobacter* metacommunity and understand how multiple sequence types simultaneously co‐occur in a flock of chickens. A combination of matrix and patch‐occupancy models was used to estimate parameters describing the competition, transmission, and mortality of each sequence type. It was found that *Campylobacter* sequence types form a strong hierarchical framework within a flock of chickens and occupied a broad spectrum of transmission–mortality trade‐offs. Upon further investigation of how biodiversity is thus maintained within the flock, it was found that the demographic capabilities of *Campylobacter*, such as mortality and transmission, could not explain the broad biodiversity of sequence types seen, suggesting that external factors such as host‐bird health and seasonality are important elements in maintaining biodiversity of *Campylobacter* sequence types.

## Graphical abstract

*Campylobacter* sequence types display a strongly hierarchical competition structure. Superior competitors at the top of the matrix outcompete the majority of those below them.

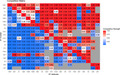

## 1 INTRODUCTION

*Campylobacter* are one of the most frequent causes of food poisoning in the UK (Strachan & Forbes, 2010), presenting an estimated £50 million direct economic burden to the UK (Tam & O’Brien, 2016). The most commonly identified route of transmission to humans is via poultry meat (EFSA Panel on Biological Hazards (BIOHAZ), 2011), with seventy‐three percent of UK supermarket chicken carcasses shown to carry the bacteria (Jorgensen et al., 2015). Whereas some foodborne pathogens, such as *Salmonella*, have been shown to proliferate primarily at the slaughterhouse (Heyndrickx et al., 2002), *Campylobacter* instead emerge and spread rapidly at the farm level (Powell et al., 2012). As a result, limiting the spread of *Campylobacter* within poultry farms has been one of the primary goals of the Food Standards Agency (FSA) across the last ten years (FSA, 2017), where attempts to date have focused on biosecurity measures (Sibanda et al., 2018), such as employing antibacterial “boot dips” at the entrance to chicken houses, and greater stress placed on farmers to practice consistent hand‐washing and facility cleaning. Since *Campylobacter* have been shown to spread from a single bird, to an entire flock, in as little as one week (Stern et al., 2001), the thinking behind such prevention methods is to minimize the chance of the bacteria entering the flock in the first instance. Such measures have proved largely ineffective (Hermans et al., 2011), prompting calls for greater study into the ecology of this microbe (Kretzschmar, 2020; Sibanda et al., 2018), in the hope of gaining insight into how it can be controlled.

Different strains of *Campylobacter* are commonly categorized by sequence type (ST); genotyping samples by multilocus sequence typing (MLST) of seven house‐keeping genes (Dingle et al., 2001; Maiden et al., 2013). Broiler flocks (birds grown for their meat) are grown for only a short time, ranging from roughly five weeks for standard flocks, to 12 weeks for organic flocks (Council of European Union, 2008). Yet despite this short window of time available for *Campylobacter* to colonize a flock, multiple STs are commonly observed simultaneously within a broiler flock (Lydekaitiene & Kudirkiene, 2020). For multiple STs to co‐occur within a flock for several weeks implies the presence of regulatory mechanisms driving the sustained biodiversity within the flock, that have not yet been identified, let alone studied in depth.

Understanding the interstrain competition mechanisms among different strains of *Campylobacter* can both aid understanding of the host–pathogen relationship, but also presents new opportunities in disease control. Understanding how certain STs may be excluded from colonizing a flock by pre‐established STs creates the opportunity for manipulation of these dynamics to reduce the incidence of certain STs. Strains of *Campylobacter* are known to vary in their pathogenic potential (Hofreuter et al., 2006), with some strains particularly effective at cell invasion (Hu & Kopecko, 1999). Introducing competitively superior strains into a transmission source presents a way to ensure that particularly pathogenic strains are unable to establish via competitive exclusion, as has been demonstrated in experimental studies (Chen & Stern, 2001). Alternatively, an understanding of these competitive frameworks presents the possibility for the use of live vaccine candidates, whereby bacterial strains that have been weakened can be used to trigger an immune response and limit pathogenic strains (Nothaft et al., 2016). While promising results in such vaccine candidates have begun to appear (Meunier et al., 2017), reliable effectiveness of these approaches requires knowledge of the underlying population dynamics. As of yet, such dynamics are not properly understood (Coward et al., 2008).

To investigate this dynamic behavior, this study utilizes two mathematical modeling approaches to query the data from a long‐term broiler–breeder flock prevalence study by Colles et al. (2015), which reports the STs isolated from individual birds within a flock across a year. A competition matrix model, such as that outlined by Ulrich et al. (2014), is used to estimate a global competition matrix, detailing the competitive outcomes of pairwise competition between STs. This matrix quantifies the likelihood of specific competitive outcomes, namely if some STs will always outcompete some other STs, or whether such competitive outcomes can have unpredictable results. More importantly, they also provide insight into the competitive hierarchy seen within the broiler microbiome. For example, one may identify a highly structured hierarchy, whereby dominant STs will always out‐compete lesser‐able STs in a gradually decreasing order of competitive advantages. Alternatively, one may identify a system of intransitive competition. Intransitive competition, or “rock‐paper‐scissors” competition, instead is defined as a system whereby loops are observed in the rank of competitive outcomes. For example, if ST A outcompetes ST B, ST B outcompetes ST C, and ST C then outcompetes ST A (Soliveres & Allan, 2018), we refer to this cyclic relationship as an intransitive triad. In such a system, there can be frequent turnover of competing organisms, as no one entity is necessarily globally superior. Intransitive competition has been shown to have far‐reaching implications for ecological stability and biodiversity, enabling species coexistence (Laird & Schamp, 2006) and promoting biodiversity (Grilli et al., 2017).

Building on this, we then use the estimated competition matrix within a discrete‐time patch‐occupancy model to simulate and explore the broader dynamics of how STs move between birds in a flock, displace one another, and capitalize on the niches presented by uncolonized birds. Patch‐occupancy models simplify a system to a series of “patches,” be it spatial units or, in our case, individual chickens, where each patch can be occupied by only one organism at a time, in our case, the dominant ST of *Campylobacter*. The turnover in occupation by different organisms is captured by a series of probabilistic transition mechanisms, which have had great success in demonstrating persistence within metacommunities (Sutherland et al., 2014), due to minimizing the assumptions placed upon the population dynamics of the system. The mechanisms that allow for sustained biodiversity in metapopulation models have been shown to primarily be the demographic factors of transmission and mortality of competing species (Hanski & Gyllenberg, 1997; May & Nowak, 1994). That is, how well a bacterium can invade a host, and how well it can remain there. In our case, we consider transmission as a measure of how many subsequent chickens will likely be challenged by the established ST in a host bird in the following time step, the outcome of such a challenge is then decided by the previously estimated competition matrix. Bacterial mortality meanwhile is considered as the probability that a dominant ST will die out in the subsequent time step, leaving the host bird susceptible to a new invading ST (not to be confused with bird mortality). By building a simulation of the system from which the data were gathered, we estimate these two specific parameters for each ST and examine how these vary between STs and how they correlate with the observed frequency of each ST.

By presenting quantified estimates into the growth, spread, and competitive ability of each individual ST, we are able to provide insight into how STs of *Campylobacter* interact with one another, both within a host chicken, and within a flock as a whole.

## 2 METHODS

### 2.1 Data

**FIGURE 1:**
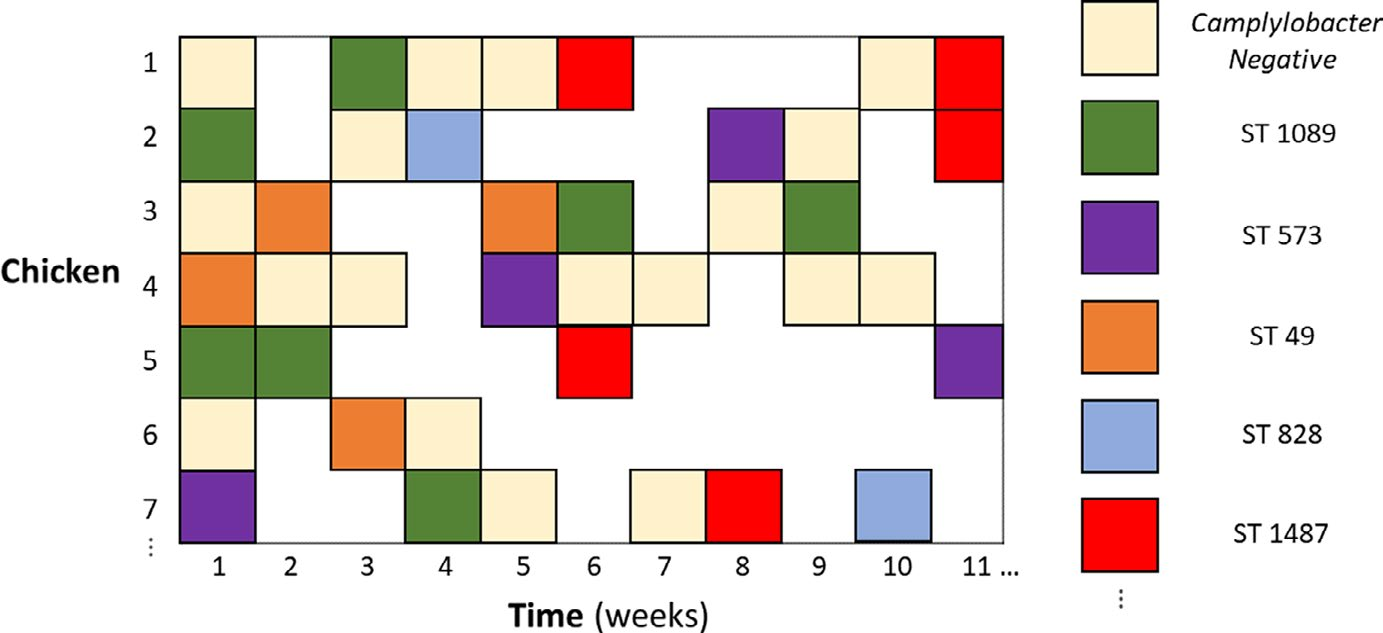
Example portion of the ST prevalence data. From a total flock of 500 broiler–breeders, 200 were labeled with leg‐rings. These 200 are captured in the rows of the data frame. Each week 75 of these birds were tested for the presence of *Campylobacter* for 51 weeks (columns). Birds were marked as either free from *Campylobacter* (marked in tan), or if found to be *Campylobacter* positive, the sequence type (ST) of the bacteria was recorded. Blank white spaces indicate where a bird was not tested for that particular week. The whole data set comprises 200 rows, 51 columns, and captures 39 distinct STs

**FIGURE 2:**
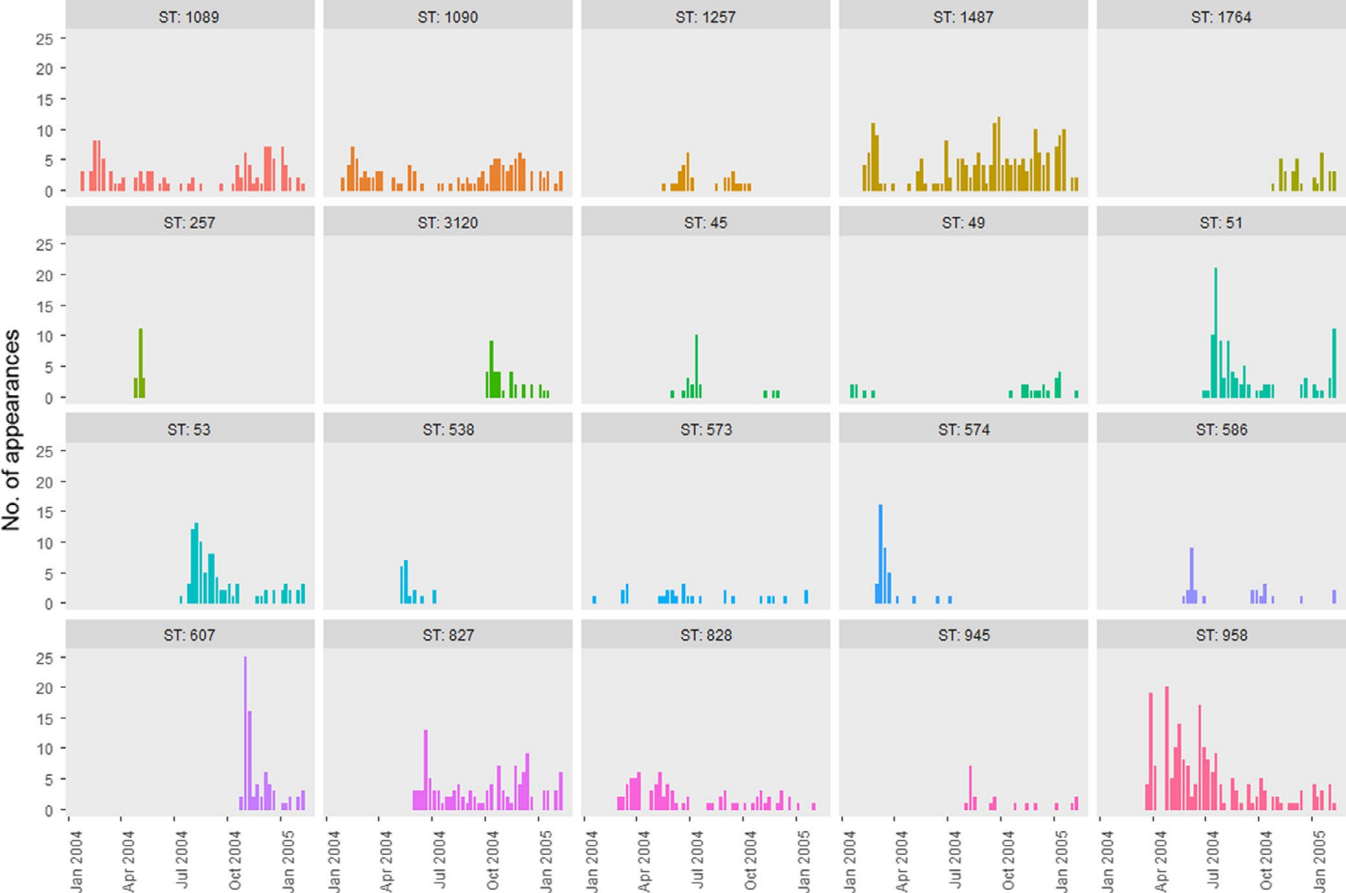
Histograms of ST prevalence across the study period reported in Colles et al. (2015). STs with less than 10 appearances are not displayed

In the original study, a flock of 500 broiler–breeders was monitored, with 200 birds labeled with leg‐rings and monitored for a total of 51 weeks. Each week, cloacal swabs were taken from a random selection of 75 of the labeled birds and tested for the presence of *Campylobacter* through standard culture methods. Positive samples were then genotyped (MLST), enabling the ST and species of the *Campylobacter* isolate to be specified. Note that, while multiple STs can occupy a host‐bird simultaneously, it is frequently observed, experimentally (Colles et al., 2019) and theoretically (Rawson et al., 2019), that a single ST will broadly dominate the gut at any given time. Hence, the sole ST recorded from a positive bird is a reflection of which STs are most dominantly expressed at that time point. Furthermore, these dominant STs in a host bird will dominate for roughly a week before being replaced by a competitor (Rawson et al., 2019). Thirty‐nine distinct STs of varying prevalence were observed across the year within the flock, 25 of *Campylobacter jejuni,* and 14 of *Campylobacter coli*. 19 of these STs appear very rarely, with less than ten total appearances in the data. Due to this limited number of data, meaningful conclusions as to their competitive abilities cannot be given, and as such, we do not consider these STs in our analysis, considering only the 20 STs for which more than ten instances of occurrence were recorded in the data. An example layout of a small portion of these data is presented in Figure 1, and the total prevalence of STs over time is displayed in Figure 2. Negative samples are not shown in Figure 2, as these data are not used for the competition matrix model. Further experimental details can be found in the original publication (Colles et al., 2015).

### 2.2 Competition matrix

We first estimate a competition matrix, detailing all pairwise competition outcomes between all STs. Formally, we define that, for a system of *n* STs, the competition matrix, *C*, is an *n* × *n* square matrix where element *C_i_*_,_ *_j_* represents the probability that ST *i* outcompetes ST *j* in a pairwise competition. By definition, the diagonal elements of *C* are equal to 1, and *C_i_*_,_ *_j_* = 1–*C_j_*_,_ *_i_*.

By using the time‐series abundance data of all STs throughout the flock, as shown in Figure 2, one may back‐infer the pairwise competitive strengths between all STs within the flock. Based upon the methods outlined by Ulrich et al. (2014), this competition matrix may be estimated by first inferring a transition matrix, *P*: an *n* × *n* square matrix where *p_i_*_,_ *_j_* represents the probability that a chicken colonized by ST *i* is instead colonized by ST *j* in the next time period. Note that this matrix *P* is not the same as the competition matrix *C*, as the observed transitions could represent the result of multiple sequential competitions between STs—the replacing ST has outcompeted not only the present occupant but also all other incoming STs.

The full mathematical methodology, as presented in Appendix 1, allows us to choose a trial competition matrix, *C*, convert this to a transition matrix, *P*, and then evaluate how well this transition matrix simulates the observed data. All that is further required is an approach by which to find the “best‐fitting” competition matrix *C*. As such, we estimate the competition matrix *C* using the equations of Appendix 1 within a Bayesian framework, using the Just Another Gibbs Sampler (JAGS) program (Plummer, 2007), a Markov chain Monte Carlo (MCMC) sampling program utilizing Gibbs sampling. Specifically, the model was called and analyzed within R by using the rjags package (Plummer et al., 2016). We considered wide, uninformative, uniform priors on the elements of *C*. Convergence was considered well‐achieved, with every element of *C*’s posterior distribution displaying a potential scale reduction factor (PSRF) <1.03, and a Monte Carlo standard error (MCSE) <5% of the standard deviation of the sample. The code used is made available at https://osf.io/3rd4e/.

Lastly, we quantify the amount of intransitivity observed from the best‐fit competition matrix *C*. While many metrics of measuring intransitivity have been proposed (Feng et al., 2020), the most suitable is generally considered to be Kendall and Babington Smith's *d_s_* (Kendall & Smith, 1940); a measure of the proportion of three‐species intransitive loops found within the competition matrix. That is, we measure the number of cyclical intransitive triads seen in the competition matrix and divide this by the total number of possible triads for a competition matrix of that size.

### 2.3 Patch‐occupancy model

The estimated competition matrix gives insight into the interactions between different *Campylobacter* STs; however, it cannot by itself answer our questions as to how biodiversity of STs is maintained within the flock. The previous metacommunity modeling studies of May and Nowak (1994) and Hanski and Gyllenberg (1997) have demonstrated that persistence can be largely managed by differences between the colonizing ability and mortality of competing organisms. As such, we estimate parameters describing the colonizing ability and mortality for each of our 20 considered STs. Figure 2 shows that some STs occur with increased frequency compared to other present STs. For example, STs 1487 and 573 both seem to persist within the flock throughout the entire recorded experimental duration, and yet ST 573 is observed in far fewer birds throughout this time. We hypothesize that differences in the demographic parameters between these STs may explain the differences in the underlying population dynamics.

A patch‐occupancy model was designed to simulate the experimental data as closely as possible. In this instance, the patches considered are the 500 chickens that make up the flock, and the STs of *Campylobacter* present are the occupying entities.

A 500 × 51 matrix is initialized, where each row denotes a specific chicken in a flock, and each column a time point (a week), so as to replicate the data structure shown in Figure 1. Element (*i*, *t*) thus records which ST, if any, has colonized chicken *i* at time *t*. The first column is initialized to match the proportion of STs recorded in the first week of the dataset in Figure 2. Each time step is then simulated in turn, to iteratively generate the subsequent 50 columns. In each time step, each established ST may be removed for the following time step with probability, *μ_i_*, the ST‐specific mortality parameter that we seek to estimate. STs that persist to the next time step then have the opportunity to infect other chickens. The number of other chickens that are challenged by this resident ST is drawn from a Poisson distribution, Pois(*λ_i_*), where *λ_i_* is a ST‐specific parameter. Borrowing from the parlance of the ecological literature, we refer to this parameter as the average “propagules released” by ST *i*. If a challenged chicken is currently uncolonized by *Campylobacter*, they then become colonized by the invading ST. If a challenged chicken is currently colonized by a different ST, this is treated as a competitive event, whereby the winner of the pairwise competition will be the occupying ST for the following time step, and the loser is removed. This outcome is decided by the median probabilities estimated in our previous model, given by the matrix *C*.

When new STs appeared for the first time in the experimental data, they are directly introduced into the patch‐occupancy model at the proportion and time step they were first observed. One exception is made for ST 49, which was unobserved for so long in the experimental data, that two specific introduction events were allowed. Appendix 2 outlines the pseudo‐code detailing this model structure. The model was programmed in R and the code is available at https://osf.io/3rd4e/.

Considering transition events on the weekly timescale provided in the original data is considered valid based upon theoretical modeling work showing that dominant STs in a host bird will dominate for roughly one week before being replaced by a competitor (Rawson et al., 2019). Much like our previous model, this provides a framework whereby a trial solution of *μ_i_* and *λ_i_* for each ST *i* can be used, and the resulting ST population dynamics can be compared against the population dynamics observed in the original data. We wish to find the values of *μ_i_* and *λ_i_* that best capture the patterns seen in Figure 2. We score a trial solution by comparing the relative proportions of ST frequency at each time step with the proportions shown in the original data.

The specific iterative framework is outlined in Appendix 2. Briefly, we first find an estimate for the average parameter values across all STs to use as an initial trial solution for each individual ST. We collapse the data to a binary state of either *Campylobacter*‐positive or *Campylobacter*‐negative and use simulated annealing to find the average *μ* and *λ* values that best simulate the data, using a scoring function defined by the absolute difference between the infection proportions in every column and every row between the model data and experimental data. This is so that the algorithm selects the parameters that also capture the frequency with which chickens may transition from being *Campylobacter*‐positive to *Campylobacter*‐negative. This provided a best‐fit solution of *μ* = 0.7, and λ = 3.2. These values were then used as initialization points for each ST‐specific parameter set (*μ_i_*, *λ_i_*), which are then iteratively adapted using genetic algorithm approaches to find the best‐fit solution. Genetic algorithms, so named for their inspiration by natural selection, generate “mutations” of the initial trial solutions, and the resulting mutations which best describe the data will in turn inform the next generation of trial solutions. A genetic algorithm of population size 200 was run for 100 iterations, using the (0.7, 3.2) estimate as a suggested population element for each specific ST.

## 3 RESULTS

**FIGURE 3:**
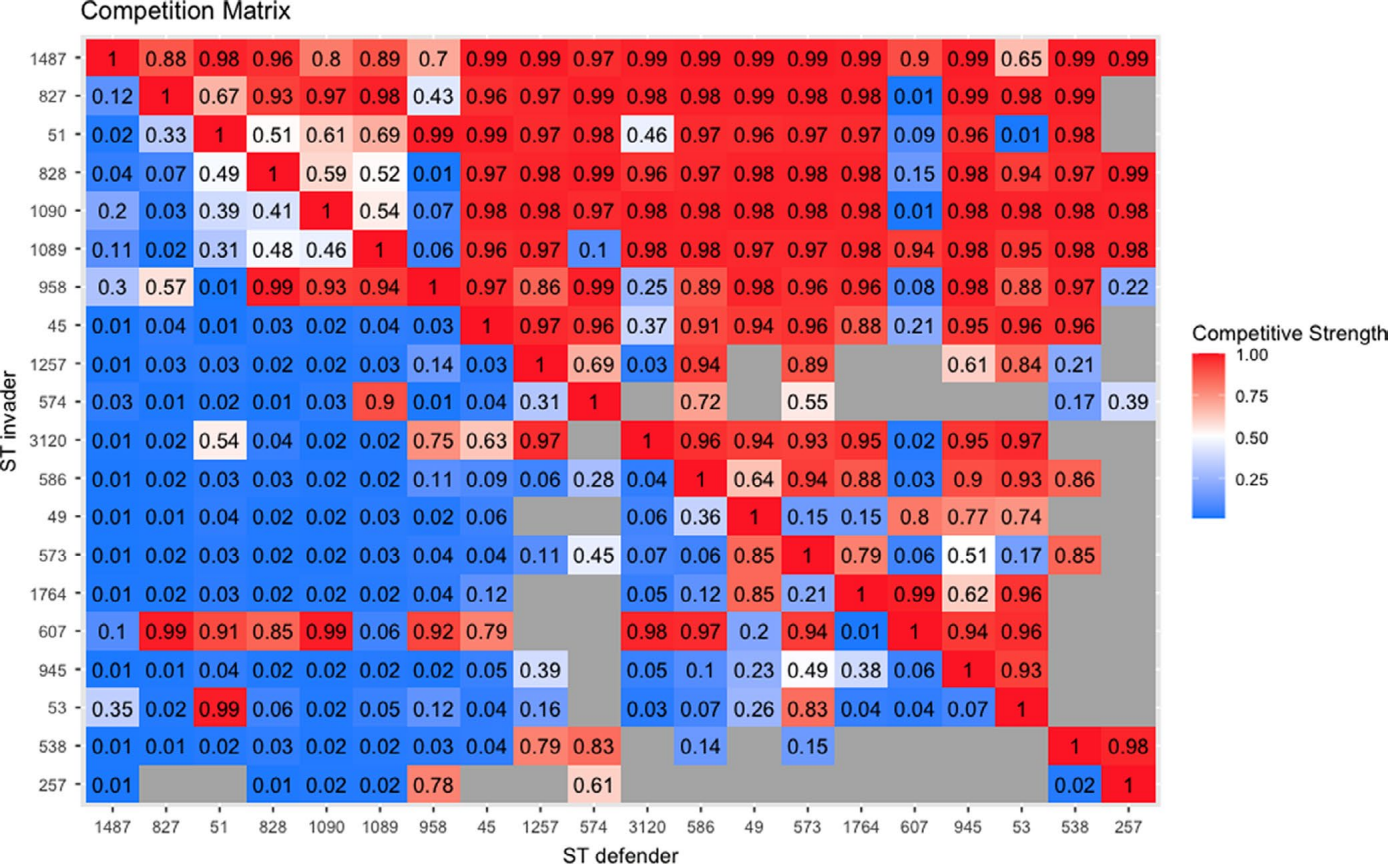
Matrix of pairwise competition strengths between *Campylobacter* STs. Element (*i*, *j*) depicts the probability that ST *i* outcompetes ST *j* in a pairwise competition. Empty gray boxes depict cases where two STs do not coexist during the experiment; thus, their competitive relationship cannot be estimated. Rows are ordered to maximize the number of values >0.5 above the diagonal. The structure reveals a strong competitive hierarchy, with the strongest competitors at the top of the matrix

Figure 3 shows the median pair‐wise competition values for all STs. 95% HDIs for each element of the competition matrix are provided in Appendix 3. STs that do not naturally co‐occur during the experiment have been represented with a gray box, as meaningful conclusions as to their competitive interactions cannot be drawn. The matrix has been re‐ordered to maximize the number of values >0.5 in the upper‐diagonal, thus showing the identified hierarchy.

A strong hierarchical structure can be observed, with STs at the top of the matrix mostly outcompeting all STs below them. Some intransitive loops can be seen within the matrix however, for example, ST 607, which is able to outcompete some STs higher up the hierarchy. When uniformly sampling the missing values of the matrix shown in Figure 3, an average of 125 intransitive triads are recorded for the competition network, compared to a hypothetical maximum of 330 for a (complete) 20 × 20 matrix, resulting in an intransitivity score of *d_s_* = 0.379 (Kendall and Babington Smith's *d_s_* (Kendall & Smith, 1940)). In comparison, on sampling 100,000 random 20 × 20 competition matrices, the lowest number of intransitive triads generated was 196; hence, our observation of only 125 triads supports a system of considerable hierarchical competition.

**FIGURE 4:**
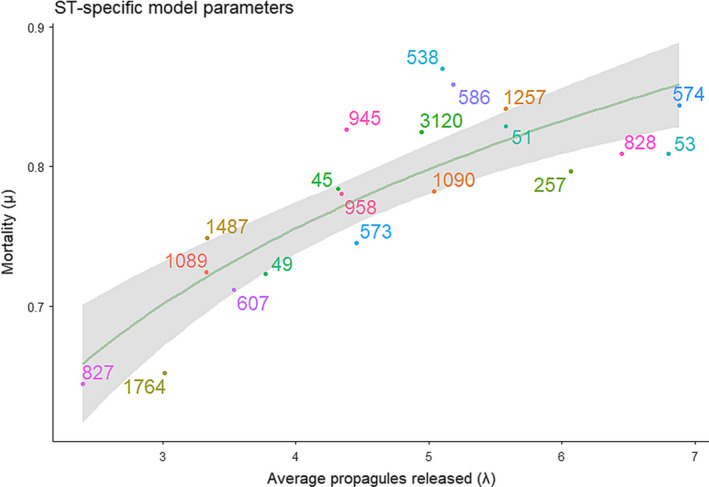
ST‐specific model parameters for patch‐occupancy model. Mortality (*μ*) depicts the probability that a ST dies out from one time‐point (a week) to the next. If the ST does not vacate a host, it releases propagules that challenge other host chickens. The average number of chickens challenged (*λ*) is a model parameter depicted on the *x*‐axis. The green line displays the statistically significant (*p* < .0001) logarithmic regression between the two variables. We see a positive trend whereby higher mortality is compensated by a greater number of propagules being released

The median competition matrix shown in Figure 3 is then utilized within the patch‐occupancy model to estimate ST‐specific transmission and mortality parameters. These parameters are displayed below in Figure 4. Mortality (*μ*) we define as the probability that an established ST will die‐out from its host bird naturally from one week to the next. To capture ST‐specific transmission effects, we report the average propagules released (*λ*), the average number of other chickens that an occupying ST will challenge for the following time step, with the outcome of these challenges decided by the above competition matrix.

The positive logarithmic trend (*p* < .0001) shows a relationship whereby STs with a higher mortality (they die out more frequently) can maintain their presence in the flock by being able to colonize more chickens.

Running the patch‐occupancy model with the best‐fit model parameters depicted in Figure 4 resulted in a wide variety of stochastic actualizations, due to the considerable impact in the resulting dynamics caused by the extinction of a ST. Nonetheless, the mean proportional ST populations across the flock captured the trends presented in Figure 2. This model validation is presented in Appendix 4.

## 4 DISCUSSION

Here, we have investigated the ecological drivers maintaining *Campylobacter* diversity within chicken flocks. By quantifying competition, transmission, and mortality parameters through two mathematical frameworks, we have highlighted the demographic differences between *Campylobacter* sequence types and shown that the metacommunity of STs operates within a strict competitive hierarchy, with some STs capable of outcompeting other STs, and hence replacing them as the dominant strain within host birds.

The competition matrix shown in Figure 3 effectively disproves the hypothesis that ST diversity may have been maintained by a system of intransitive competition, as very few intransitive triads were found within the system. Intransitive loops have been shown to theoretically support coexistence of many competing organisms, dependent on growth rate differences and intransitive cycle length (Gallien et al., 2017). Despite the wealth of theoretical work surrounding the impact of intransitive competition, real‐world evidence of such systems is lacking. An experimental study searching for such effects across five different taxonomic groups by Soliveres et al. (2018) was unable to find strong evidence of intransitivity in any of their studies other than experiments with mosses and found zero intransitive triads in their bacterial experiment. As such, our inability to identify clear signs of intransitivity is unsurprising. Only STs 53, 607, and 3120 showed clear evidence of being able to outcompete STs higher in the hierarchy. All three of these STs appear to have remained prevalent in the flock from their point of entry to the end of the experiment time, possibly suggesting that STs that are able to form an intransitive loop may be more capable of invading and persisting in the flock.

Within this competitive hierarchy, we also show that the magnitude of the respective competitive probabilities is relatively large. In the upper diagonal of Figure 3 ,values are greater than 0.9, suggesting that the competitively superior STs not only outcompete a vast number of other STs, but that they outcompete these other STs decisively, winning competitive interactions over 90% of the time in most instances. This is in line with experimental studies into interstrain competition, with El‐Shibiny et al. (2007) demonstrating how a strain of *Campylobacter* is not only able to outcompete a multitude of other strains, but to do so repeatedly in multiple experiments.

As shown in Appendix 3, the uncertainty range of the values of the competition matrix, *C*, are tightest among the most frequently observed STs, primarily the strongest competitors. As such the only noteworthy uncertainty is found for the competition value between two STs that very rarely co‐exist in the flock. For these cases, the posterior estimates are only slightly converted from their uniform priors and as such display median competition values close to 0.5. Given that the high majority of posterior densities are tightly converged, only the median estimates were used in defining the transition model.

This evidence of a clear competitive hierarchy further stresses how specific mechanisms must underpin the observed maintenance of biodiversity of *Campylobacter* STs. Under such competitive conditions, biodiversity of a metacommunity has been shown to be feasibly maintained by trade‐offs between transmission and mortality (Bonsall et al., 2004; May & Nowak, 1994; Scheffer & van Nes, 2006). Under such a system, the co‐occurrence of multiple STs can be explained by competitively strong STs displaying high mortality rates, namely that after replacing a resident ST, they naturally die out from the host quickly. Alternatively, their transmission ability may be compromised such that, although they may be very effective competitors, they are unable to proliferate as fast as other STs, and thus may not challenge a high number of other chickens from one week to the next. Likewise, a competitively weak ST, such as ST 53 in Figure 3, may not be able to withstand competition from incoming STs, but is able to persist in the flock by challenging a higher number of chickens each week (high number of propagules released) and surviving within these host birds for a longer period of time (low mortality). The patch‐occupancy model presented was designed to specifically quantify these mortality and mean propagule release parameters and is presented in Figure 4.

Figure 4 shows that all STs can be placed somewhere within a life‐history trade‐off. In general, STs displaying high mortality may persist in the environment by releasing a higher number of average propagules and vice‐versa. May and Nowak (1994) theoretically showed that for a newly emerging entity into a community to successfully invade a metacommunity, and to then persist, they need to fill a yet unrepresented area of this transmission–mortality spectrum. That is, to persist, they need to have no close neighbors in the plot of Figure 4. This may be demonstrated by STs 827 and 53. Both STs can be seen from Figure 2 to appear within the flock mid‐way through the time span and to then successfully persist through to the end of the experiment. Both of these STs can also be seen from Figure 4 to be outliers on the transmission–mortality spectrum, with ST 827 having the lowest mortality and lowest mean propagules released of all observed STs, and ST 53 having the second highest number of mean propagules released. As a further interesting contrast, competitive ability does not appear to have influenced this, as ST 53 is one of the weakest competitors in the metacommunity, and ST 827 is one of the strongest, as shown in Figure 3.

However, this mechanism alone has historically been unable to account for the vast amount of sustained biodiversity observed in nature. Building on the theoretical findings of May and Nowak (1994), Bonsall et al. (2004) demonstrated that species within a hierarchical competition structure, competing for the same resource, may co‐exist by clustering into “life‐history guilds.” Competitively strong species may simultaneously co‐exist by sharing similar demographic parameters. At the same time, competitively weaker species will also persist in the environment, by also sharing similar demographic capabilities with one another. Scheffer and van Nes (2006) highlighted the same result, concluding that newly emergent species would only persist in the environment if either (i) they were significantly competitively superior to all existing species, or (ii) if they were similar enough to existing species, both competitively and demographically, so as to exist within this particular life‐history guild niche. Our results however do not show evidence of such ecological guilds.

Figure 4 shows that, while STs do form a life‐history trade‐off, STs appear in a broadly even distribution across this mortality–propagule trade‐off. Furthermore, some STs that appear to be demographically similar vary greatly in their competitive ability and respective population dynamics. From Figure 2, we can broadly delineate STs by four distinct dynamic profiles: a ST may either persist in a flock or die out, and it may exist at high frequency or low frequency. It was assumed that one could characterize these four distinct dynamic profiles by their competition, average propagule release, and mortality parameters, and yet no such pattern has been found in this study. For example, the STs 257, 574, 45, and 1257 could all be characterized as appearing in high frequency, before then dying‐out. Yet despite these similar dynamical behaviors, all STs place broadly across the competition–propagule–mortality spectrum, with no common trends in their placement. Likewise, STs 586, 573, and 945 could all be categorized as persisting in the flock, though recovered at low frequency, and yet all three STs are found in broadly different placements in Figures 3 and 4. In general, STs that appear in high frequency appear to correlate with higher competitive potential in Figure 3, though no such trend can be associated with persistence.

Since these STs do not demonstrate the guild‐assemblage “clumping” structure in Figure 4 (shown by Bonsall et al. (2004) to be necessary for biodiversity maintenance in this instance), it suggests that some other mechanism must be enabling the co‐occurrence and persistence of *Campylobacter* STs. Based upon the broader wealth of investigations into *Campylobacter* dynamics, we can posit three potential hypotheses driving these clearly seen differences in population dynamics between STs:

- Host‐bird variability. It has been shown in numerous patch‐occupancy systems that patch quality (meaning that some patches are “easier” to colonize than others) can have a tremendous impact on the overall population dynamics, having even greater impact than differences between how patches are connected (Poniatowski et al., 2018). Yu and Wilson (2001) theoretically showed that while differences in life‐history trade‐offs were necessary for co‐existence, significant heterogeneity in patch quality or density was necessary to support a large number of species. Such patch variation also made it possible for newly emergent species to persist even if the species was inferior in both competitive and colonization ability. In our context, variation in patch quality and density would translate to host birds varying in their response to bacterial challenge, with some chickens “easier” to colonize than others. Indeed, through Bayesian transition models we have shown using this same data set in Rawson et al. (2020) that a flock contains a mixture of birds that are highly resilient to bacterial challenge and highly susceptible birds that operate as “super shedders.” These super shedders are consistently being colonized by a variety of *Campylobacter* STs with high turnover. Poor individual bird health and welfare has been previously shown to correlate with a reduced immune response, with measures such as stocking density (Gomes et al., 2014), food withdrawal, and heat stress (Burkholder et al., 2008) all contributing to increased *Campylobacter* colonization. Yu and Wilson (2001) directly showed that the host‐bird variation seen in Rawson et al. (2020) removes the need for newly emerging STs to be sufficiently similar to persist in the flock. This further supports the idea that the proliferation of *Campylobacter* in a flock is influenced primarily by the individual birds.
- Seasonal variation. Broiler flock colonization by *Campylobacter* has been well‐documented to follow a seasonal trend (Jore et al., 2010), with flocks more likely to become colonized in the warmer summer months than the winter. The data behind this modeling study were gathered over 51 weeks, January 2004 to January 2005, so would plausibly have been impacted by seasonal variation. The original study examining the impact of local environmental variables on the data set we have considered (Colles et al., 2015) (and subsequent Bayesian transition analyses (Rawson et al., 2020)), were unable to identify any temporal trend within the total *Campylobacter* prevalence; however, the *Campylobacter coli* STs did appear less frequently during the summer. It is thus plausible that seasonal variation may have impacted the population dynamics of the occupying STs in the flock via some yet‐unidentified mechanism. An example of this may be seen by comparing the population dynamics of STs 53 and 574. Both STs occupy a similar placement in the propagule–mortality spectrum of Figure 4, and yet, despite ST 574 being more competitively able than ST 53, ST 574 does not persist in the flock, while ST 53 does. One possible explanation for this is that ST first appeared within the flock in July, while ST 574 appeared in February.
- Stochasticity. While our patch‐occupancy model is a probabilistic one, the mechanisms by which a metacommunity of *Campylobacter* STs persist is determined by a number of random events. The events of a ST first entering the flock, chickens ingesting colonized feces, and of then establishing themselves within the microbiome all encompass a wide number of stochastic events which could change the resulting population dynamics. Coward et al. (2008) showed that attempts to replicate population dynamics of *Campylobacter* within broilers were largely unsuccessful, even in the most simple cases of just two competing strains. They posited that this was likely due to “founder effects,” small variations in population level at first inoculation which could have large consequences for the flock‐wide population dynamics. We have previously shown this effect through a series of stochastic differential equations in Rawson et al. (2019), whereby a variety of overall population dynamics can be observed dependent on stochastic events when the population of a *Campylobacter* ST is very low. Likewise, upon running the patch‐occupancy model for the estimated parameters presented in the results, some STs would persist in some actualizations, but not others. Thus, attempting to characterize some dynamical profiles by mortality and transmission parameters may not be possible as our experiment displays only one dynamic outcome of many possible ones.

Indeed, further to this point, when the patch‐occupancy model is run with the best‐fit parameters presented in Figure 4, a wide variety of outcomes is seen. STs may persevere with ease in one actualization, or quickly die out in another. Each actualization however is only ever dominated by a small handful of STs, and the model is rarely able to capture stable coexistence of as many STs as seen in the experimental data. While the mean dynamics seen broadly match those seen in the experimental data (thus being the best‐fit), the regularity of extinctions observed in simulations further supports the conclusion that the model as it currently stands is unable to explain the observed biodiversity, further stressing one of the above three options as an explanation.

One important caveat to this work must be stressed. Since broiler flocks are slaughtered anywhere from 5 to 11 weeks of age, longitudinal studies into the *Campylobacter* population dynamics are not possible, birds are not alive for long enough for us to observe long‐term dynamics from which to extract parameter estimates. As such, these experimental data were gathered from a flock of broiler–breeders, the birds that lay the eggs that become broiler flocks. As we have discussed above, host bird factors may have significant implications for the overarching population dynamics of the microbiome, meaning that these estimated parameters could plausibly be different in commercial broiler flocks. Broiler and breeder flocks are kept under slightly different housing conditions and diet provisions (Leeson & Summers, 2010), and breeder flocks have also been shown to shed smaller amounts of *Campylobacter* than commercial broilers (Cox et al., 2002). Since this study has focused on investigating *Campylobacter*‐specific factors, our conclusions remain relevant to commercial broiler flocks, namely that the population dynamics remain deeply susceptible to impact from a variety of factors, such as season and host bird health. We also note that we do not explicitly consider the impact of the saturation of the surrounding environment with *Campylobacter* as a factor driving colonization patterns. While we have considered the impact of environment colonization in our previous theoretical modeling works (Rawson et al., 2019), the impact of such factors was found to be small in comparison to host‐bird factors.

The primary finding of this work highlights how the life‐history trade‐offs we have identified fail to provide an explanation for the persistence and co‐occurrence of multiple *Campylobacter* STs. This further supports the notion that suppressing and controlling outbreaks of *Campylobacter* cannot be achieved through bio‐security alone and reflects calls for a “One Health” (Gölz et al., 2014) approach, whereby further understanding is needed of how *Campylobacter* and broilers interact and affect each other. We have shown that demographic advantages alone cannot determine which STs of *Campylobacter* will come to dominate a flock of chickens and that it may instead come down to a ST being in the right place at the right time, or rather, the right chicken in the right season.

## CONFLICT OF INTEREST

The author declares that the research was conducted in the absence of any commercial or financial relationships that could be construed as a potential conflict of interest.

## AUTHOR CONTRIBUTIONS

**Thomas Rawson:** Conceptualization (equal); Data curation (lead); Formal analysis (lead); Investigation (lead); Methodology (equal); Project administration (equal); Software (lead); Visualization (lead); Writing—original draft (lead); Writing—review and editing (equal). **Frances Colles:** Project administration (equal); Resources (lead); Supervision (equal); Writing—review and editing (equal). **J. Christopher D. Terry:** Conceptualization (equal); Supervision (equal); Writing—review and editing (equal). **Michael Bonsall:** Conceptualization (equal); Project administration (equal); Supervision (equal); Writing—review and editing (equal).

## Supporting information

Fig A1

Fig A2

Appendix S1

## Data Availability

All model code used is made available at https://osf.io/3rd4e/, https://doi.org/10.17605/OSF.IO/3RD4E

## References

[ece38651-bib-0001] Bonsall, M. B. , Jansen, V. A. , & Hassell, M. P. (2004). Life history trade‐offs assemble ecological guilds. Science, 306(5693), 111–382. 15459391 10.1126/science.1100680

[ece38651-bib-0002] Burkholder, K. , Thompson, K. , Einstein, M. , Applegate, T. , & Patterson, J. (2008). Influence of stressors on normal intestinal microbiota, intestinal morphology, and susceptibility to Salmonella enteritidis colonization in broilers. Poultry Science, 87, 1734–1741. 10.3382/ps.2008-00107 18753440

[ece38651-bib-0003] Chen, H.‐C. , & Stern, N. J. (2001). Competitive exclusion of heterologous *Campylobacter* spp. in chicks. Applied and Environmental Microbiology, 67(2), 848–851. 11157253 10.1128/AEM.67.2.848-851.2001 PMC92657

[ece38651-bib-0004] Colles, F. M. , McCarthy, N. D. , Bliss, C. M. , Layton, R. , & Maiden, M. C. (2015). The long‐term dynamics of *Campylobacter* colonizing a free‐range broiler breeder flock: an observational study. Environmental Microbiology, 17(4), 938–946. 25588789 10.1111/1462-2920.12415 PMC4390391

[ece38651-bib-0005] Colles, F. M. , Preston, S. G. , Barfod, K. K. , Flammer, P. G. , Maiden, M. C. , & Smith, A. L. (2019). Parallel sequencing of pora reveals a complex pattern of *Campylobacter* genotypes that diapers between broiler and broiler breeder chickens. Scientific Reports, 9(1), 1–13. 30996225 10.1038/s41598-019-42207-9 PMC6470227

[ece38651-bib-0006] Council of European Union (2008). Commission Regulation (EC) No 889/2008 of 5 September 2008 laying down detailed rules for the implementation of Council Regulation (EC) No 834/2007 on organic production and labelling of organic products with regard to organic production, labelling and control. https://eur‐lex.europa.eu/legal‐content/EN/TXT/?uri=CELEX%3A02008R0889‐20210101

[ece38651-bib-0007] Coward, C. , van Diemen, P. M. , Conlan, A. J. , Gog, J. R. , Stevens, M. P. , Jones, M. A. , & Maskell, D. J. (2008). Competing isogenic *Campylobacter* strains exhibit variable population structures in vivo. Applied and Environmental Microbiology, 74(12), 3857–3867. 18424530 10.1128/AEM.02835-07 PMC2446568

[ece38651-bib-0008] Cox, N. , Stern, N. , Musgrove, M. , Bailey, J. , Craven, S. , Cray, P. , Buhr, R. , & Hiett, K. (2002). Prevalence and level of *Campylobacter* in commercial broiler breeders (parents) and broilers. Journal of Applied Poultry Research, 11(2), 187–190.

[ece38651-bib-0009] Dingle, K. , Colles, F. , Wareing, D. , Ure, R. , Fox, A. , Bolton, F. , Bootsma, H. , Willems, R. , Urwin, R. , & Maiden, M. (2001). Multilocus sequence typing system for *Campylobacter jejuni* . Journal of Clinical Microbiology, 39(1), 14–23. 11136741 10.1128/JCM.39.1.14-23.2001 PMC87672

[ece38651-bib-0010] EFSA Panel on Biological Hazards (BIOHAZ) (2011). Scientific Opinion on *Campylobacter* in broiler meat production: control options and performance objectives and/or targets at different stages of the food chain. EFSA Journal, 9(4), 2105. 10.2903/j.efsa.2011.2105 PMC13129689 42078736

[ece38651-bib-0011] El‐Shibiny, A. , Connerton, P. , & Connerton, I. (2007). *Campylobacter* succession in broiler chickens. Veterinary Microbiology, 125(3–4), 323–332. 17628357 10.1016/j.vetmic.2007.05.023

[ece38651-bib-0012] Feng, Y. , Soliveres, S. , Allan, E. , Rosenbaum, B. , Wagg, C. , Tabi, A. , De Luca, E. , Eisenhauer, N. , Schmid, B. , Weigelt, A. , Weisser, W. , Roscher, C. , & Fischer, M. (2020). Inferring competitive outcomes, ranks and intransitivity from empirical data: A comparison of different methods. Methods in Ecology and Evolution, 11(1), 117–128.

[ece38651-bib-0013] FSA (2010). The Joint Government and Industry Target to Reduce *Campylobacter* in UK Produced Chickens by 2015. FSA.

[ece38651-bib-0014] Gallien, L. , Zimmermann, N. E. , Levine, J. M. , & Adler, P. B. (2017). The effects of intransitive competition on coexistence. Ecology Letters, 20(7), 791–800. 28547799 10.1111/ele.12775

[ece38651-bib-0015] Gölz, G. , Rosner, B. , Hofreuter, D. , Josenhans, C. , Kreienbrock, L. , Löwenstein, A. , Schielke, A. , Stark, K. , Suerbaum, S. , Wieler, L. H. , Alter, T. (2014). Relevance of *Campylobacter* to public health—the need for a One Health approach. International Journal of Medical Microbiology, 304(7), 817–823. 25266744 10.1016/j.ijmm.2014.08.015

[ece38651-bib-0016] Gomes, A. , Quinteiro‐Filho, W. M. , Ribeiro, A. , Ferraz‐de Paula, V. , Pinheiro, M. , Baskeville, E. , Akamine, A. T. , Astolfi‐Ferreira, C. S. , Ferreira, A. J. P. , & Palermo‐Neto, J. (2014). Overcrowding stress decreases macrophage activity and increases Salmonella enteritidis invasion in broiler chickens. Avian Pathology, 43(1), 82–90. 24350836 10.1080/03079457.2013.874006

[ece38651-bib-0017] Grilli, J. , Barabás, G. , Michalska‐Smith, M. J. , & Allesina, S. (2017). Higher‐order interactions stabilize dynamics in competitive network models. Nature, 548(7666), 210–213. 28746307 10.1038/nature23273

[ece38651-bib-0018] Hanski, I. , & Gyllenberg, M. (1997). Uniting two general patterns in the distribution of species. Science, 275(5298), 397–400. 8994039 10.1126/science.275.5298.397

[ece38651-bib-0019] Hermans, D. , Van Deun, K. , Messens, W. , Martel, A. , Van Immerseel, F. , Haesebrouck, F. , Rasschaert, G. , Heyndrickx, M. , & Pasmans, F. (2011). *Campylobacter* control in poultry by current intervention measures ineffective: urgent need for intensified fundamental research. Veterinary Microbiology, 152(3–4), 219–228. 21482043 10.1016/j.vetmic.2011.03.010

[ece38651-bib-0020] Heyndrickx, M. , Vandekerchove, D. , Herman, L. , Rollier, I. , Grijspeerdt, K. , & De Zutter, L. (2002). Routes for Salmonella contamination of poultry meat: epidemiological study from hatchery to slaughterhouse. Epidemiology & Infection, 429 129(2), 253–265. 10.1017/s0950268802007380 PMC2869884 12403101

[ece38651-bib-0021] Hofreuter, D. , Tsai, J. , Watson, R. O. , Novik, V. , Altman, B. , Benitez, M. , Clark, C. , Perbost, C. , Jarvie, T. , Du, L. , Galan, J. E. (2006). Unique features of a highly pathogenic *Campylobacter* jejuni strain. Infection and Immunity, 74(8), 4694–4707. 16861657 10.1128/IAI.00210-06 PMC1539605

[ece38651-bib-0022] Hu, L. , & Kopecko, D. J. (1999). *Campylobacter jejuni* 81–176 associates with microtubules and dynein during invasion of human intestinal cells. Infection and Immunity, 67(8), 4171–4182. 10417189 10.1128/iai.67.8.4171-4182.1999 PMC96722

[ece38651-bib-0023] Jore, S. , Viljugrein, H. , Brun, E. , Heier, B. , Borck, B. , Ethelberg, S. , Hakkinen, M. , Kuusi, M. , Reiersen, J. , Hansson, I. , Olsson Engvall, E. , Løfdahl, M. , Wagenaar, J. A. , van Pelt, W. , & Hofshagen, M. (2010). Trends in *Campylobacter* incidence in broilers and humans in six European countries, 1997–2007. Preventive Veterinary Medicine, 93(1), 33–41. 19837471 10.1016/j.prevetmed.2009.09.015

[ece38651-bib-0024] Jorgensen, F. , Madden, R. H. , Arnold, E. , Charlett, A. , & Elviss, N. C. (2015). FSA Project FS241044 ‐ Survey report ‐ A Microbiological survey of *Campylobacter* contamination in fresh whole UK produced chilled chickens at retail sale (2014‐15). Public Health England (PHE), Food Standards Agency (FSA). https://www.food.gov.uk/sites/default/files/media/document/Final%20Report%20for%20FS241044%20Campylobacter%20Retail%20survey.pdf

[ece38651-bib-0025] Kendall, M. G. , & Smith, B. B. (1940). On the method of paired comparisons. Biometrika, 31(3/4), 324–345.

[ece38651-bib-0026] Kretzschmar, M. (2020). Disease modeling for public health: added value, challenges, and institutional constraints. Journal of Public Health Policy, 41(1), 39–51. 31780754 10.1057/s41271-019-00206-0 PMC7041603

[ece38651-bib-0027] Laird, R. A. , & Schamp, B. S. (2006). Competitive intransitivity promotes species coexistence. The American Naturalist, 168(2), 182–193. 10.1086/506259 16874628

[ece38651-bib-0028] Leeson, S. , & Summers, J. D. (2010). Broiler breeder production. Nottingham University Press.

[ece38651-bib-0029] Lydekaitiene, V. L. , & Kudirkiene, E. (2020). Prevalence and genetic diversity of *C. jejuni* isolated from broilers and their environment using flaA‐RFLP typing and MLST analysis. Annals of Animal Science, 20(2), 485–501. 10.2478/aoas-2020-0008

[ece38651-bib-0030] Maiden, M. C. , Van Rensburg, M. J. J. , Bray, J. E. , Earle, S. G. , Ford, S. A. , Jolley, K. A. , & McCarthy, N. D. (2013). MLST revisited: the gene‐by‐gene approach to bacterial genomics. Nature Reviews Microbiology, 11(10), 728–736. 23979428 10.1038/nrmicro3093 PMC3980634

[ece38651-bib-0031] May, R. M. , & Nowak, M. A. (1994). Superinfection, metapopulation dynamics, and the evolution of diversity. Journal of Theoretical Biology, 170(1), 95–114. 7967636 10.1006/jtbi.1994.1171

[ece38651-bib-0032] Meunier, M. , Guyard‐Nicodème, M. , Vigouroux, E. , Poezevara, T. , Beven, V. , Quesne, S. , Bigault, L. , Amelot, M. , Dory, D. , & Chemaly, M. (2017). Promising new vaccine candidates against *Campylobacter* in broilers. PLoS One, 12(11), e0188472. 29176789 10.1371/journal.pone.0188472 PMC5703506

[ece38651-bib-0033] Nothaft, H. , Davis, B. , Lock, Y. Y. , Perez‐Munoz, M. E. , Vinogradov, E. , Walter, J. , Coros, C. , & Szymanski, C. M. (2016). Engineering the *Campylobacter jejuni* N‐glycan to create an effective chicken vaccine. Scientific Reports, 6(1), 1–12. 27221144 10.1038/srep26511 PMC4879521

[ece38651-bib-0034] Plummer, M. (2007). JAGS: A program for analysis of Bayesian graphical models using Gibbs sampling. http://mcmc‐jags.sourceforge.net/

[ece38651-bib-0035] Plummer, M. , Stukalov, A. , & Denwood, M. (2016). rjags: Bayesian graphical models using MCMC. R package version. http://CRAN.R‐project.org/package=rjags

[ece38651-bib-0036] Poniatowski, D. , Stuhldreher, G. , Löffler, F. , & Fartmann, T. (2018). Patch occupancy of grassland specialists: Habitat quality matters more than habitat connectivity. Biological Conservation, 225, 237–244.

[ece38651-bib-0037] Powell, L. , Lawes, J. , Clifton‐Hadley, F. , Rodgers, J. , Harris, K. , Evans, S. , & Vidal, A. (2012). The prevalence of *Campylobacter* spp. in broiler flocks and on broiler carcases, and the risks associated with highly contaminated carcases. Epidemiology & Infection, 140(12), 2233–2246. 22336562 10.1017/S0950268812000040 PMC9152337

[ece38651-bib-0038] Rawson, T. , Colles, F. M. , Terry, J. C. D. , & Bonsall, M. B. (2021). Mechanisms of biodiversity between *Campylobacter* sequence types in a flock of broiler‐breeder chickens. bioRxiv. 10.1002/ece3.8651 PMC8928907 35342550

[ece38651-bib-0039] Rawson, T. , Dawkins, M. S. , & Bonsall, M. B. (2019). A mathematical model of *Campylobacter* dynamics within a broiler flock. Frontiers in Microbiology, 10, 1940. 31497006 10.3389/fmicb.2019.01940 PMC6712969

[ece38651-bib-0040] Rawson, T. , Paton, R. S. , Colles, F. M. , Maiden, M. C. , Dawkins, M. S. , & Bonsall, M. B. (2020). A mathematical modeling approach to uncover factors influencing the spread of *Campylobacter* in a flock of broiler‐breeder chickens. Frontiers in Microbiology, 11, 2481. 10.3389/fmicb.2020.576646 PMC7655537 33193192

[ece38651-bib-0041] Scheffer, M. , & van Nes, E. H. (2006). Self‐organized similarity, the evolutionary emergence of groups of similar species. Proceedings of the National Academy of Sciences, 103(16), 6230–6235. 10.1073/pnas.0508024103 PMC1458860 16585519

[ece38651-bib-0042] Sibanda, N. , McKenna, A. , Richmond, A. , Ricke, S. C. , Callaway, T. , Stratakos, A. C. , Gundogdu, O. , & Corcionivoschi, N. (2018). A review of the effect of management practices on *Campylobacter* prevalence in poultry farms. Frontiers in Microbiology, 9, 2002. 30197638 10.3389/fmicb.2018.02002 PMC6117471

[ece38651-bib-0043] Soliveres, S. , & Allan, E. (2018). Everything 476 you always wanted to know about intransitive competition but were afraid to ask. Journal of Ecology, 106(3), 807–814.

[ece38651-bib-0044] Soliveres, S. , Lehmann, A. , Boch, S. , Altermatt, F. , Carrara, F. , Crowther, T. W. , Delgado‐Baquerizo, M. , Kempel, A. , Maynard, D. S. , Rillig, M. C. , Singh, B. K. , Trivedi, P. , & Allan, E. (2018). Intransitive competition is common across five major taxonomic groups and is driven by productivity, competitive rank and functional traits. Journal of Ecology, 106(3), 852–864.

[ece38651-bib-0045] Stern, N. J. , Cox, N. A. , Musgrove, M. T. , & Park, C. (2001). Incidence and levels of *Campylobacter* in broilers after exposure to an inoculated seeder bird. Journal of Applied Poultry Research, 10(4), 315–318.

[ece38651-bib-0046] Strachan, N. J. , & Forbes, K. J. (2010). The growing UK epidemic of human campylobacteriosis. The Lancet, 376(9742), 665–667. 10.1016/S0140-6736(10)60708-8 20663545

[ece38651-bib-0047] Sutherland, C. , Elston, D. , & Lambin, X. (2014). A demographic, spatially explicit patch occupancy model of metapopulation dynamics and persistence. Ecology, 95(11), 3149–3160.

[ece38651-bib-0048] Tam, C. C. , & O’Brien, S. J. (2016). Economic cost of *campylobacter*, norovirus and rotavirus disease in the United Kingdom. PLoS One, 11(2), e0138526. 26828435 10.1371/journal.pone.0138526 PMC4735491

[ece38651-bib-0049] Ulrich, W. , Soliveres, S. , Kryszewski, W. , Maestre, F. T. , & Gotelli, N. J. (2014). Matrix models for quantifying competitive intransitivity from species abundance data. Oikos, 123(9), 1057–1070. 25914427 10.1111/oik.01217 PMC4407980

[ece38651-bib-0050] Yu, D. W. , & Wilson, H. B. (2001). The competition‐colonization trade‐off is dead; long live the competition‐colonization trade‐off. The American Naturalist, 158(1), 49–63. 10.1086/320865 18707314

