# Supplementary figures and images for: Mechanisms of biodiversity between Campylobacter sequence types in a flock of broiler–breeder chickens
Source: Ecol Evol. 2022 Mar 6;12(3):e8651. doi: 10.1002/ece3.8651 (PMC8928907; doi:10.1002/ece3.8651)

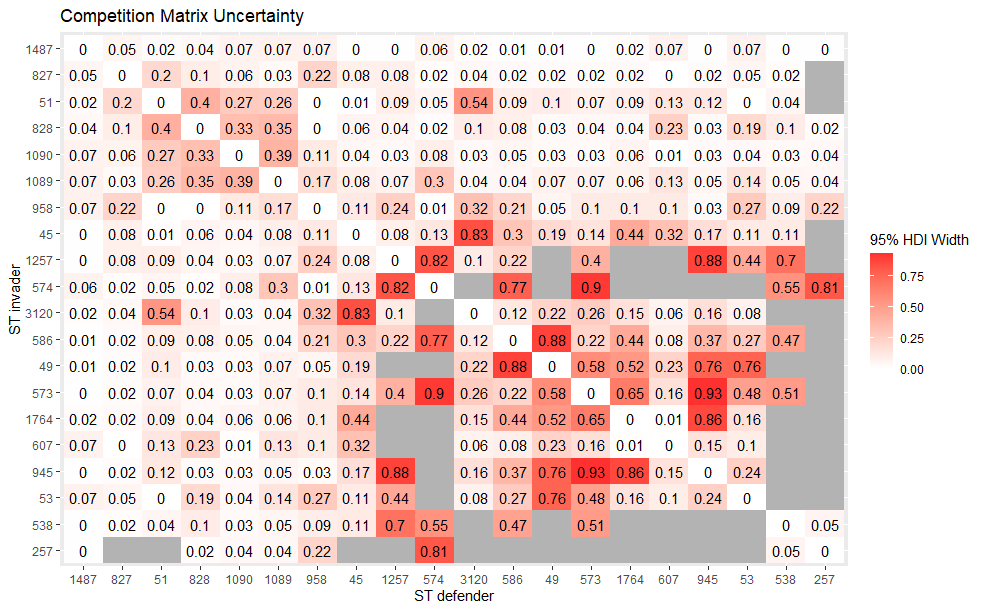

Supplement: Supplementary file 1 — Fig A1 [file ECE3-12-e8651-s002.tiff]

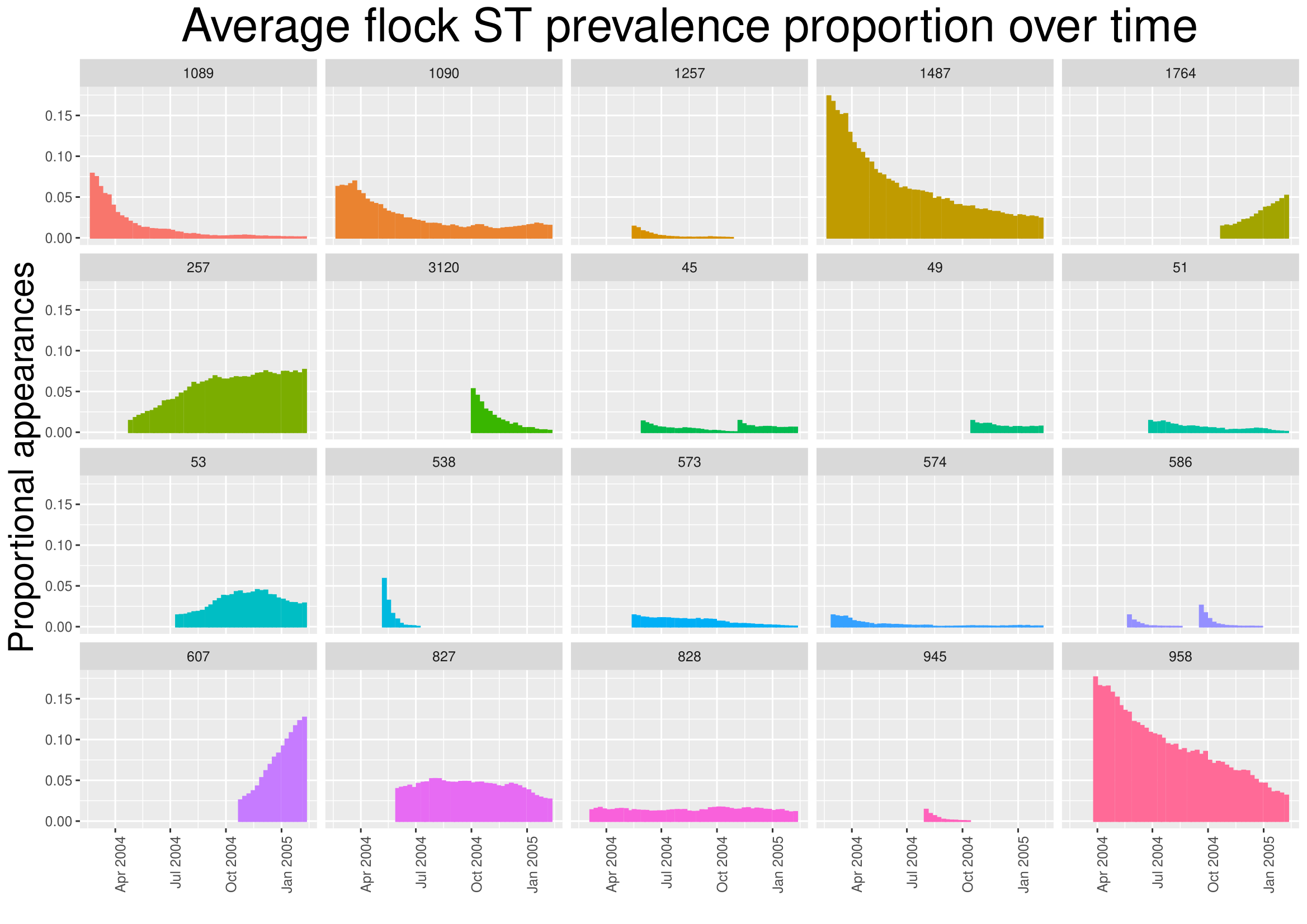

Supplement: Supplementary file 2 — Fig A2 [file ECE3-12-e8651-s001.tiff]
