# Supplementary material for: Mechanisms of biodiversity between Campylobacter sequence types in a flock of broiler–breeder chickens
Source: Ecol Evol. 2022 Mar 6;12(3):e8651. doi: 10.1002/ece3.8651 (PMC8928907; doi:10.1002/ece3.8651)
Supplement: Supplementary file 3 — Appendix S1 [file ECE3-12-e8651-s003.pdf]

# A Appendices

## A.1 Appendix 1 - Calculating the competition matrix

We first estimate a competition matrix, detailing all pairwise competition outcomes between all STs. Formally, we define that, for a system of $n$ STs, the competition matrix, $C$, is an $n \times n$ square matrix where element $C_{i,j}$ represents the probability that ST $i$ out-competes ST $j$ in a pairwise competition. By definition, the diagonal elements of $C$ are equal to 1, and $C_{i,j} = 1 - C_{j,i}$.

By using the time-series abundance data of all STs throughout the flock, as shown in Figure 2, one may back-infer the pairwise competitive strengths between all STs within the flock. Based upon the methods outlined by Ulrich et al. (2014), this competition matrix may be estimated by first inferring a transition matrix, $P$: an $n \times n$ square matrix where $P_{i,j}$ represents the probability that a chicken colonised by ST $i$ is instead colonised by ST $j$ in the next time period. Note that this matrix $P$ is not the same as the competition matrix $C$, as the observed transitions could represent the result of multiple sequential competitions between STs - the replacing ST has not only outcompeted the present occupant, but also all other incoming STs.

To estimate this transition matrix, $P$, consider an $n \times 51$ frequency matrix $A$, where element $A_{i,t}$ denotes the number of chickens that ST $i$ was isolated from at time $t$, and where $n$ is the number of distinct STs. This matrix is directly built from our data, where element $A_{i,t}$ can be seen as the 'No. of appearances' of ST $i$ in week $t$ from Figure 2. This frequency matrix is then related to our transition matrix, $P$, via the equation;

$$PA_t = A_{t+1} \tag{1}$$

where $A_t$ is a column vector of $n$ elements, reporting the abundance of all STs at time $t$. This provides a method by which to estimate $P$ by choosing the matrix $P$ that best fits equation (1). Homogeneous mixing of STs is assumed, however, another assumption is made in equation (1) that all STs are present and are capable of appearing at each time point. This is not representative of biological reality. We see from Figure 2 that some STs do not appear in the flock until later in the experiment, and while it could plausibly be being out-competed in every prior instance, it is more plausible that the ST has simply not yet infected the flock. As such, we adapt equation (1) by also implementing a binary-filled 'presence' matrix $Z$, an $n \times 51$ matrix, where element $Z_{i,j}$ is either 0 or 1, denoting whether or not ST $i$ is present in the flock at time $j$. i.e. when a ST is not observed within a flock in a particular week, we do not consider it's impact on that week's transition dynamics.

If ST $i$ is isolated in the data at time $t$, we mark it as present in matrix $Z$ for times $t$ through to $t+3$, to account for the possibility of a ST being reduced to low levels, not captured in the data. This three week window was determined from our earlier numerical simulations (Rawson et al., 2019), showing the average duration for which a low-level "non-dominant"

ST might persist within a host. We rewrite equation (1) as:

$$(PA_t) \odot Z_{t+1} = A_{t+1} \tag{2}$$

where $Z_{t+1}$ is the $(t+1)^{\text{th}}$ column of $Z$, and $\odot$ is the Hadamard (element-wise) product. In essence, $Z$ simply acts as a switching mechanism, to switch off the possibility of transitions to a ST that has not yet emerged. This approach carries multiple benefits. Primarily, the transition matrix now represents the transition probabilities for a flock where all STs are present simultaneously. This inference allows more of the dataset to be utilised, without having to divide our experimental data into multiple regions of different sized matrix calculations. A possible limitation to this approach is that it allows inference of competitive outcomes between STs that do not appear at the same time in the original dataset. i.e. it can infer based on the growth abilities of a ST at a later time how it would fare against a ST from an earlier time. While this inference is useful, these limited instances are not experimentally verifiable. As such, we do not display these few "assumed" competitive strengths in our results, to avoid confusion.

Once the best fitting $P$ to equation (2) has been found, we may use this $P$ to estimate the associated competition matrix $C$. Ulrich et al. (2014) presents such a methodology whereby, assuming homogeneous mixing, the transition matrix $P$ and the competition matrix $C$ are linked by the relationship:

$$P_{i,j} = P(1,\ldots,n)\,[j \to i] = \frac{1}{n-1}C_{i,j} + \frac{1}{n-1}\sum_{k=1,k\neq i,j}^{n} C_{j,k}P(1,\ldots,k-1,k+1,\ldots,n)\,[j \to i] \tag{3}$$

for $i \neq j$, and

$$P_{i,i} = \prod_{k=1,k\neq i}^{n} C_{i,k} \tag{4}$$

where the range of summation in (4) is calculated across the subset considered in the notation $P(1,\ldots,n)$. Heuristically, one considers the transition probabilities as the proportional outcomes of all possible competitive interactions. In a four-species system, equations (3) and (4) would define:

$$P_{i,j} = \frac{1}{3}C_{i,j} + \frac{1}{3}\left(\frac{1}{2}C_{i,j}C_{j,k} + \frac{1}{2}C_{i,j}C_{j,k}C_{j,l}\right) + \frac{1}{3}\left(\frac{1}{2}C_{i,j}C_{j,l} + \frac{1}{2}C_{i,j}C_{j,l}C_{j,k}\right).$$

In small systems, the probability of successful transition for each ST could be directly calculated as the proportional

outcome of all possible competitive interactions as given in equations (3) and (4). However, for our system of 20 STs this is computationally impossible, as the size of equation (3) will rapidly balloon for such a large system. Instead we therefore used the approximation approach of Ulrich et al. (2014), utilising the geometric mean of the associated competition values:

$$P_{i,j} \approx \frac{1}{m-1} C_{i,j} \sum_{k=0}^{m-2} \bar{x}^k. \tag{5}$$

where $\bar{x}$ is the geometric mean of the competition values associated with $P_{i,j}$.

Upon testing, we found that this approximation was found to estimate a randomly drawn $20 \times 20$ test competition matrix with a mean value error < 0.001.

The above methodology allows us to choose a trial competition matrix, $C$, convert this to a transition matrix, $P$ via equation (5), and then evaluate how well this transition matrix simulates the observed data, $A$, via equation (2). All that is required now is an approach by which to find the “best" competition matrix $C$. As such, we estimate the competition matrix $C$ using the above equations within a Bayesian framework, using the Just Another Gibbs Sampler (JAGS) program (Plummer, 2007), a Markov chain Monte Carlo (MCMC) sampling program utilising Gibbs sampling. Specifically the model was called and analysed within R by using the `rjags` package (Plummer et al., 2016). We considered wide, uninformative, uniform priors on the elements of $C$. Convergence was considered well-achieved, with every element of $C$'s posterior distribution displaying a potential scale reduction factor (PSRF) < 1.03, and a Monte Carlo standard error (MCSE) less than 5% of the standard deviation of the sample. The code used is made available at https://osf.io/3rd4e/.

Lastly, we quantify the amount of intransitivity observed from the best-fit competition matrix $C$. While many metrics of measuring intransitivity have been proposed (Feng et al., 2020), the most suitable is generally considered to be Kendall and Babington Smith's $d_s$ (Kendall and Smith, 1940); a measure of the proportion of three-species intransitive loops found within the competition matrix. i.e. we measure the number of cyclical intransitive triads seen in the competition matrix, and divide this by the total number of possible triads for a competition matrix of that size.

## A.2 Appendix 2 - Patch-occupancy model pseudo-code

**Algorithm 1:** Patch-occupancy model pseudo code

```
Initialise chickens with STs in proportion to very first timestep in experimental data.
for every timestep do
    Prepare placeholder vector for current timestep, equal to previous timestep.
    for every chicken do
        if chicken currently colonised then
            Record currently occupying ST s.
            Draw random number x from uniform distribution U(0,1).
            if x < μ_s then
                Remove ST s from chicken in placeholder vector.
            else
                ST s will challenge other chickens:
                Draw random number y from Pois(λ_s).
                Add y to a running tally, Y_s, of how many other chickens will be challenged by ST s.
            end
        end
    end
    for every ST, s do
        for j ← 1 to Y_s do
            Randomly select a chicken, c, to be challenged by ST s.
            if c not colonised then
                Chicken c is now colonised by ST s in placeholder vector.
            else
                Record currently occupying ST, r.
                Draw a random number z from uniform distribution U(0,1).
                if z < C_{s,r} then
                    ST s replaces ST r in placeholder vector.
                else
                    ST r remains in chicken c in placeholder vector.
                end
            end
        end
    end
    Placeholder vector is assigned as frequency vector for current timestep. Move to subsequent timestep.
end
```

### A.3 Appendix 3 - Competition Matrix Uncertainty

Figure 3 of the manuscript presents the competition matrix, where the elements plotted are the median of the Bayesian posterior distributions for each element. Table A1 below presents the median values, and the upper and lower values of the 95% HDIs of the posterior. Diagonals are not presented as they are set equal to 1, and the lower diagonal is not presented, as these are set by subtracting their upper diagonal counterparts from 1. For example, the HDI of C[13,4] is equivalent to 1 - the HDI of C[4,13]. Figure A1 presents the range of the HDIs (upper - lower) in the same format as Figure 3. For the great majority of values, this is $< 0.1$, save for circumstances when two strains only co-exist very briefly in the experiment duration - strains which are then quickly outcompeted. Uncertainty is very small for competitively superior strains, as would be expected, as more data is available for these strains for which to calculate their competitive capabilities.

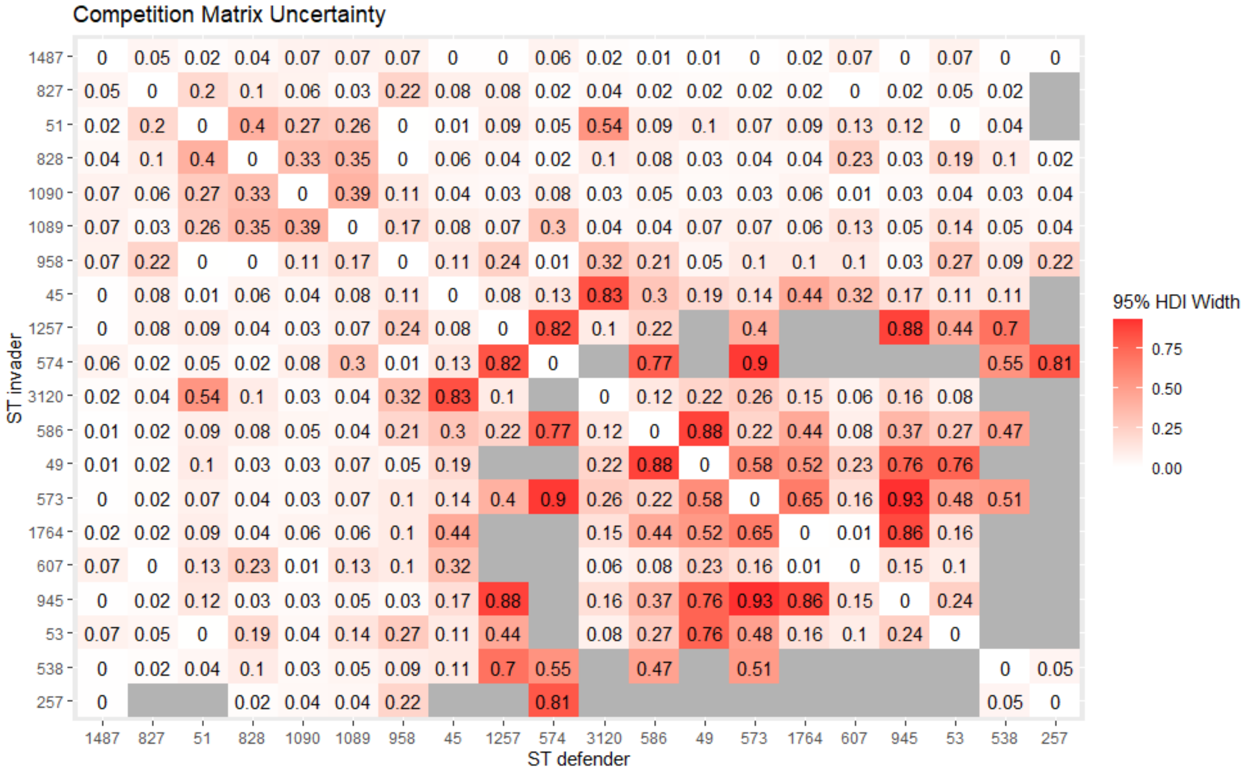


**Figure A1.** HDI width magnitudes for the Bayesian posterior distributions of each element of the competition matrix, ordered as presented in Figure 3.

**Table A1.** Median values and upper/lower 95% HDI limits of the posterior distributions of all parameters within the competition matrix, $C$, as presented in Figure 3.

| Parameter | ST Invader | ST Defender | Median | Upper | Lower |
|---|---|---|---|---|---|
| C[1\|2] | 1487 | 1090 | 0.802 | 0.835 | 0.769 |
| C[1\|3] | 1487 | 45 | 0.989 | 0.99 | 0.985 |
| C[1\|4] | 1487 | 51 | 0.982 | 0.99 | 0.97 |
| C[1\|5] | 1487 | 827 | 0.885 | 0.909 | 0.858 |
| C[1\|6] | 1487 | 1257 | 0.989 | 0.99 | 0.985 |
| C[1\|7] | 1487 | 573 | 0.989 | 0.99 | 0.985 |
| C[1\|8] | 1487 | 49 | 0.989 | 0.99 | 0.985 |
| C[1\|9] | 1487 | 1089 | 0.892 | 0.929 | 0.857 |
| C[1\|10] | 1487 | 574 | 0.966 | 0.99 | 0.929 |
| C[1\|11] | 1487 | 958 | 0.699 | 0.735 | 0.666 |
| C[1\|12] | 1487 | 53 | 0.654 | 0.69 | 0.621 |
| C[1\|13] | 1487 | 828 | 0.962 | 0.979 | 0.943 |
| C[1\|14] | 1487 | 257 | 0.989 | 0.99 | 0.986 |
| C[1\|15] | 1487 | 3120 | 0.986 | 0.99 | 0.973 |
| C[1\|16] | 1487 | 945 | 0.989 | 0.99 | 0.986 |
| C[1\|17] | 1487 | 607 | 0.903 | 0.939 | 0.865 |
| C[1\|18] | 1487 | 1764 | 0.986 | 0.99 | 0.972 |
| C[1\|19] | 1487 | 586 | 0.989 | 0.99 | 0.985 |
| C[1\|20] | 1487 | 538 | 0.989 | 0.99 | 0.985 |
| C[2\|3] | 1090 | 45 | 0.979 | 0.99 | 0.949 |
| C[2\|4] | 1090 | 51 | 0.386 | 0.52 | 0.252 |
| C[2\|5] | 1090 | 827 | 0.033 | 0.074 | 0.01 |
| C[2\|6] | 1090 | 1257 | 0.982 | 0.99 | 0.956 |
| C[2\|7] | 1090 | 573 | 0.983 | 0.99 | 0.959 |
| C[2\|8] | 1090 | 49 | 0.983 | 0.99 | 0.962 |
| C[2\|9] | 1090 | 1089 | 0.544 | 0.739 | 0.348 |
| C[2\|10] | 1090 | 574 | 0.969 | 0.99 | 0.906 |
| C[2\|11] | 1090 | 958 | 0.066 | 0.126 | 0.016 |
| C[2\|12] | 1090 | 53 | 0.982 | 0.99 | 0.955 |

| Parameter | ST Invader | ST Defender | Median | Upper | Lower |
|---|---|---|---|---|---|
| C[2\|13] | 1090 | 828 | 0.408 | 0.575 | 0.248 |
| C[2\|14] | 1090 | 257 | 0.981 | 0.99 | 0.954 |
| C[2\|15] | 1090 | 3120 | 0.984 | 0.99 | 0.964 |
| C[2\|16] | 1090 | 945 | 0.984 | 0.99 | 0.964 |
| C[2\|17] | 1090 | 607 | 0.011 | 0.017 | 0.01 |
| C[2\|18] | 1090 | 1764 | 0.977 | 0.99 | 0.932 |
| C[2\|19] | 1090 | 586 | 0.979 | 0.99 | 0.943 |
| C[2\|20] | 1090 | 538 | 0.983 | 0.99 | 0.958 |
| C[3\|4] | 45 | 51 | 0.012 | 0.018 | 0.01 |
| C[3\|5] | 45 | 827 | 0.036 | 0.09 | 0.01 |
| C[3\|6] | 45 | 1257 | 0.973 | 0.99 | 0.912 |
| C[3\|7] | 45 | 573 | 0.958 | 0.99 | 0.85 |
| C[3\|8] | 45 | 49 | 0.945 | 0.99 | 0.8 |
| C[3\|9] | 45 | 1089 | 0.035 | 0.095 | 0.01 |
| C[3\|10] | 45 | 574 | 0.957 | 0.99 | 0.864 |
| C[3\|11] | 45 | 958 | 0.029 | 0.122 | 0.01 |
| C[3\|12] | 45 | 53 | 0.964 | 0.99 | 0.883 |
| C[3\|13] | 45 | 828 | 0.027 | 0.068 | 0.01 |
| C[3\|14] | 45 | 257 | 1 | 1 | 1 |
| C[3\|15] | 45 | 3120 | 0.368 | 0.843 | 0.01 |
| C[3\|16] | 45 | 945 | 0.95 | 0.99 | 0.822 |
| C[3\|17] | 45 | 607 | 0.213 | 0.378 | 0.058 |
| C[3\|18] | 45 | 1764 | 0.883 | 0.99 | 0.554 |
| C[3\|19] | 45 | 586 | 0.91 | 0.99 | 0.693 |
| C[3\|20] | 45 | 538 | 0.964 | 0.99 | 0.879 |
| C[4\|5] | 51 | 827 | 0.328 | 0.426 | 0.229 |
| C[4\|6] | 51 | 1257 | 0.967 | 0.99 | 0.903 |
| C[4\|7] | 51 | 573 | 0.972 | 0.99 | 0.916 |
| C[4\|8] | 51 | 49 | 0.964 | 0.99 | 0.889 |
| C[4\|9] | 51 | 1089 | 0.687 | 0.814 | 0.557 |
| C[4\|10] | 51 | 574 | 0.977 | 0.99 | 0.94 |

| Parameter | ST Invader | ST Defender | Median | Upper | Lower |
|---|---|---|---|---|---|
| C[4\|11] | 51 | 958 | 0.99 | 0.99 | 0.988 |
| C[4\|12] | 51 | 53 | 0.011 | 0.012 | 0.01 |
| C[4\|13] | 51 | 828 | 0.513 | 0.716 | 0.311 |
| C[4\|14] | 51 | 257 | 1 | 1 | 1 |
| C[4\|15] | 51 | 3120 | 0.459 | 0.736 | 0.197 |
| C[4\|16] | 51 | 945 | 0.96 | 0.99 | 0.869 |
| C[4\|17] | 51 | 607 | 0.091 | 0.163 | 0.034 |
| C[4\|18] | 51 | 1764 | 0.969 | 0.99 | 0.902 |
| C[4\|19] | 51 | 586 | 0.969 | 0.99 | 0.904 |
| C[4\|20] | 51 | 538 | 0.979 | 0.99 | 0.946 |
| C[5\|6] | 827 | 1257 | 0.972 | 0.99 | 0.912 |
| C[5\|7] | 827 | 573 | 0.985 | 0.99 | 0.967 |
| C[5\|8] | 827 | 49 | 0.986 | 0.99 | 0.971 |
| C[5\|9] | 827 | 1089 | 0.984 | 0.99 | 0.961 |
| C[5\|10] | 827 | 574 | 0.986 | 0.99 | 0.974 |
| C[5\|11] | 827 | 958 | 0.431 | 0.541 | 0.319 |
| C[5\|12] | 827 | 53 | 0.979 | 0.99 | 0.943 |
| C[5\|13] | 827 | 828 | 0.933 | 0.974 | 0.879 |
| C[5\|14] | 827 | 257 | 1 | 1 | 1 |
| C[5\|15] | 827 | 3120 | 0.98 | 0.99 | 0.952 |
| C[5\|16] | 827 | 945 | 0.986 | 0.99 | 0.971 |
| C[5\|17] | 827 | 607 | 0.01 | 0.011 | 0.01 |
| C[5\|18] | 827 | 1764 | 0.985 | 0.99 | 0.966 |
| C[5\|19] | 827 | 586 | 0.985 | 0.99 | 0.966 |
| C[5\|20] | 827 | 538 | 0.986 | 0.99 | 0.974 |
| C[6\|7] | 1257 | 573 | 0.892 | 0.99 | 0.59 |
| C[6\|8] | 1257 | 49 | 1 | 1 | 1 |
| C[6\|9] | 1257 | 1089 | 0.026 | 0.075 | 0.01 |
| C[6\|10] | 1257 | 574 | 0.685 | 0.99 | 0.172 |
| C[6\|11] | 1257 | 958 | 0.143 | 0.25 | 0.01 |
| C[6\|12] | 1257 | 53 | 0.842 | 0.99 | 0.547 |

| Parameter | ST Invader | ST Defender | Median | Upper | Lower |
|---|---|---|---|---|---|
| C[6\|13] | 1257 | 828 | 0.02 | 0.055 | 0.01 |
| C[6\|14] | 1257 | 257 | 1 | 1 | 1 |
| C[6\|15] | 1257 | 3120 | 0.034 | 0.115 | 0.01 |
| C[6\|16] | 1257 | 945 | 0.609 | 0.99 | 0.113 |
| C[6\|17] | 1257 | 607 | 1 | 1 | 1 |
| C[6\|18] | 1257 | 1764 | 1 | 1 | 1 |
| C[6\|19] | 1257 | 586 | 0.938 | 0.99 | 0.766 |
| C[6\|20] | 1257 | 538 | 0.212 | 0.706 | 0.01 |
| C[7\|8] | 573 | 49 | 0.846 | 0.99 | 0.414 |
| C[7\|9] | 573 | 1089 | 0.028 | 0.085 | 0.01 |
| C[7\|10] | 573 | 574 | 0.449 | 0.914 | 0.01 |
| C[7\|11] | 573 | 958 | 0.04 | 0.113 | 0.01 |
| C[7\|12] | 573 | 53 | 0.169 | 0.492 | 0.01 |
| C[7\|13] | 573 | 828 | 0.018 | 0.046 | 0.01 |
| C[7\|14] | 573 | 257 | 1 | 1 | 1 |
| C[7\|15] | 573 | 3120 | 0.073 | 0.271 | 0.01 |
| C[7\|16] | 573 | 945 | 0.505 | 0.94 | 0.014 |
| C[7\|17] | 573 | 607 | 0.06 | 0.17 | 0.01 |
| C[7\|18] | 573 | 1764 | 0.79 | 0.99 | 0.338 |
| C[7\|19] | 573 | 586 | 0.061 | 0.229 | 0.01 |
| C[7\|20] | 573 | 538 | 0.853 | 0.99 | 0.48 |
| C[8\|9] | 49 | 1089 | 0.027 | 0.083 | 0.01 |
| C[8\|10] | 49 | 574 | 1 | 1 | 1 |
| C[8\|11] | 49 | 958 | 0.022 | 0.057 | 0.01 |
| C[8\|12] | 49 | 53 | 0.744 | 0.99 | 0.232 |
| C[8\|13] | 49 | 828 | 0.018 | 0.043 | 0.01 |
| C[8\|14] | 49 | 257 | 1 | 1 | 1 |
| C[8\|15] | 49 | 3120 | 0.065 | 0.227 | 0.01 |
| C[8\|16] | 49 | 945 | 0.766 | 0.99 | 0.233 |
| C[8\|17] | 49 | 607 | 0.805 | 0.913 | 0.686 |
| C[8\|18] | 49 | 1764 | 0.154 | 0.529 | 0.01 |

| Parameter | ST Invader | ST Defender | Median | Upper | Lower |
|---|---|---|---|---|---|
| C[8\|19] | 49 | 586 | 0.356 | 0.889 | 0.01 |
| C[8\|20] | 49 | 538 | 1 | 1 | 1 |
| C[9\|10] | 1089 | 574 | 0.1 | 0.306 | 0.01 |
| C[9\|11] | 1089 | 958 | 0.056 | 0.179 | 0.01 |
| C[9\|12] | 1089 | 53 | 0.951 | 0.99 | 0.849 |
| C[9\|13] | 1089 | 828 | 0.483 | 0.655 | 0.302 |
| C[9\|14] | 1089 | 257 | 0.979 | 0.99 | 0.948 |
| C[9\|15] | 1089 | 3120 | 0.981 | 0.99 | 0.951 |
| C[9\|16] | 1089 | 945 | 0.978 | 0.99 | 0.94 |
| C[9\|17] | 1089 | 607 | 0.938 | 0.99 | 0.856 |
| C[9\|18] | 1089 | 1764 | 0.976 | 0.99 | 0.928 |
| C[9\|19] | 1089 | 586 | 0.981 | 0.99 | 0.949 |
| C[9\|20] | 1089 | 538 | 0.977 | 0.99 | 0.937 |
| C[10\|11] | 574 | 958 | 0.013 | 0.023 | 0.01 |
| C[10\|12] | 574 | 53 | 1 | 1 | 1 |
| C[10\|13] | 574 | 828 | 0.014 | 0.028 | 0.01 |
| C[10\|14] | 574 | 257 | 0.393 | 0.82 | 0.012 |
| C[10\|15] | 574 | 3120 | 1 | 1 | 1 |
| C[10\|16] | 574 | 945 | 1 | 1 | 1 |
| C[10\|17] | 574 | 607 | 1 | 1 | 1 |
| C[10\|18] | 574 | 1764 | 1 | 1 | 1 |
| C[10\|19] | 574 | 586 | 0.716 | 0.99 | 0.216 |
| C[10\|20] | 574 | 538 | 0.165 | 0.556 | 0.01 |
| C[11\|12] | 958 | 53 | 0.877 | 0.99 | 0.722 |
| C[11\|13] | 958 | 828 | 0.99 | 0.99 | 0.989 |
| C[11\|14] | 958 | 257 | 0.216 | 0.327 | 0.111 |
| C[11\|15] | 958 | 3120 | 0.247 | 0.423 | 0.102 |
| C[11\|16] | 958 | 945 | 0.984 | 0.99 | 0.962 |
| C[11\|17] | 958 | 607 | 0.08 | 0.137 | 0.034 |
| C[11\|18] | 958 | 1764 | 0.963 | 0.99 | 0.885 |
| C[11\|19] | 958 | 586 | 0.888 | 0.99 | 0.779 |

| Parameter | ST Invader | ST Defender | Median | Upper | Lower |
|---|---|---|---|---|---|
| C[11\|20] | 958 | 538 | 0.97 | 0.99 | 0.899 |
| C[12\|13] | 53 | 828 | 0.062 | 0.201 | 0.01 |
| C[12\|14] | 53 | 257 | 1 | 1 | 1 |
| C[12\|15] | 53 | 3120 | 0.027 | 0.085 | 0.01 |
| C[12\|16] | 53 | 945 | 0.069 | 0.254 | 0.01 |
| C[12\|17] | 53 | 607 | 0.036 | 0.106 | 0.01 |
| C[12\|18] | 53 | 1764 | 0.045 | 0.166 | 0.01 |
| C[12\|19] | 53 | 586 | 0.072 | 0.277 | 0.01 |
| C[12\|20] | 53 | 538 | 1 | 1 | 1 |
| C[13\|14] | 828 | 257 | 0.986 | 0.99 | 0.973 |
| C[13\|15] | 828 | 3120 | 0.956 | 0.99 | 0.893 |
| C[13\|16] | 828 | 945 | 0.982 | 0.99 | 0.958 |
| C[13\|17] | 828 | 607 | 0.145 | 0.268 | 0.035 |
| C[13\|18] | 828 | 1764 | 0.981 | 0.99 | 0.952 |
| C[13\|19] | 828 | 586 | 0.972 | 0.99 | 0.912 |
| C[13\|20] | 828 | 538 | 0.967 | 0.99 | 0.889 |
| C[14\|15] | 257 | 3120 | 1 | 1 | 1 |
| C[14\|16] | 257 | 945 | 1 | 1 | 1 |
| C[14\|17] | 257 | 607 | 1 | 1 | 1 |
| C[14\|18] | 257 | 1764 | 1 | 1 | 1 |
| C[14\|19] | 257 | 586 | 1 | 1 | 1 |
| C[14\|20] | 257 | 538 | 0.021 | 0.065 | 0.01 |
| C[15\|16] | 3120 | 945 | 0.951 | 0.99 | 0.828 |
| C[15\|17] | 3120 | 607 | 0.022 | 0.073 | 0.01 |
| C[15\|18] | 3120 | 1764 | 0.954 | 0.99 | 0.836 |
| C[15\|19] | 3120 | 586 | 0.963 | 0.99 | 0.87 |
| C[15\|20] | 3120 | 538 | 1 | 1 | 1 |
| C[16\|17] | 945 | 607 | 0.055 | 0.159 | 0.01 |
| C[16\|18] | 945 | 1764 | 0.383 | 0.866 | 0.01 |
| C[16\|19] | 945 | 586 | 0.097 | 0.38 | 0.01 |
| C[16\|20] | 945 | 538 | 1 | 1 | 1 |

| Parameter | ST Invader | ST Defender | Median | Upper | Lower |
|---|---|---|---|---|---|
| C[17\|18] | 607 | 1764 | 0.011 | 0.017 | 0.01 |
| C[17\|19] | 607 | 586 | 0.968 | 0.99 | 0.907 |
| C[17\|20] | 607 | 538 | 1 | 1 | 1 |
| C[18\|19] | 1764 | 586 | 0.118 | 0.449 | 0.01 |
| C[18\|20] | 1764 | 538 | 1 | 1 | 1 |
| C[19\|20] | 586 | 538 | 0.857 | 0.99 | 0.515 |

## A.4 Appendix 4 - Model Validation

The best-fit patch occupancy model parameters presented in Figure 3 result in a wide variety of stochastic evaluations: in some instances a ST may flourish, while in other simulations it may quickly die out, due to random chance. The model was run 100 times, and we present the mean proportion of STs in the flock across these 100 simulations below in Figure A2. We present the results in the same format as Figure 2 to allow direct comparison.

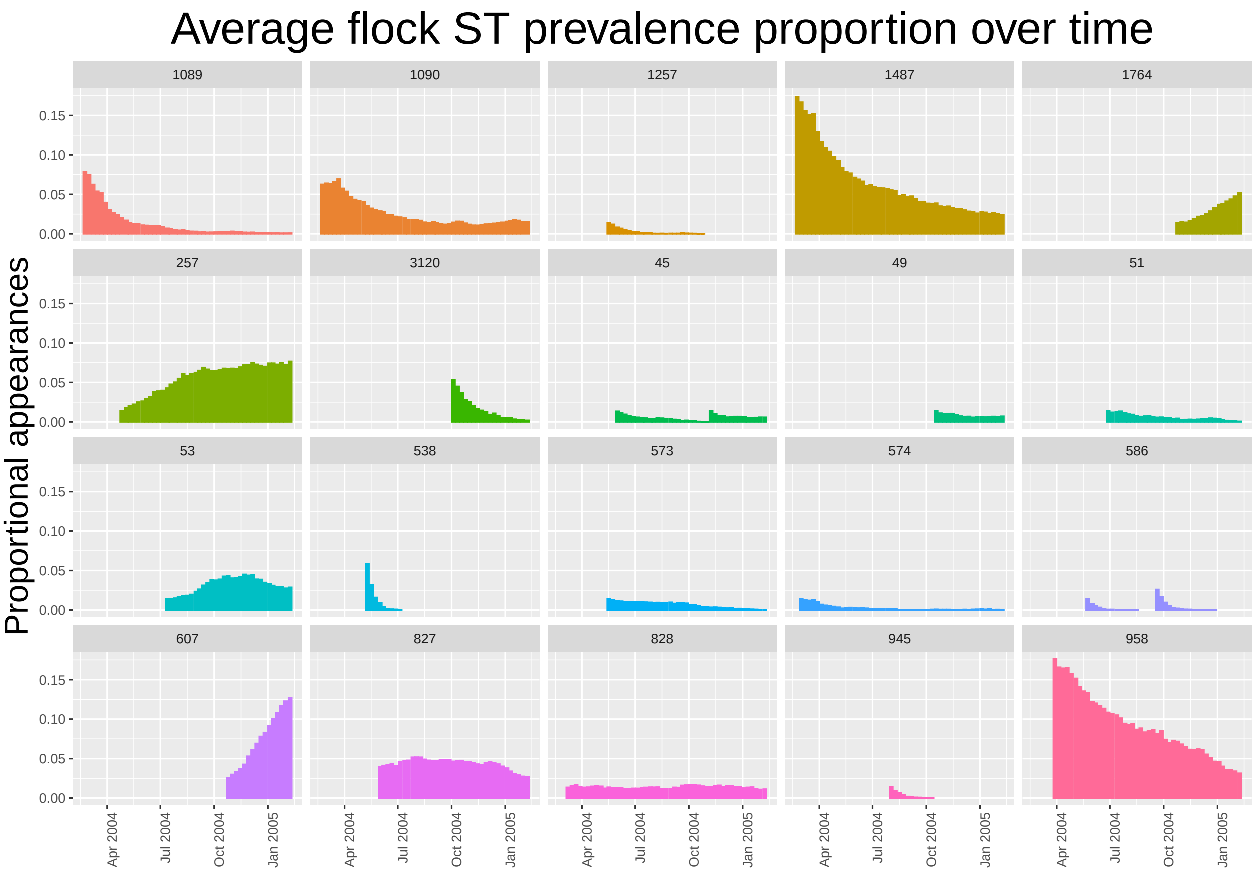


**Figure A2.** Mean proportion of STs simulated over 100 simulations of the patch occupancy model.

Note that the trend seen above of slowly decreasing STs should not be interpreted as an accurate depiction of ST dynamics, rather a representation of probability of persistence to that point in time. ST 1089 for example, is less likely to be maintained in actualisations than ST 1487, but the magnitude of prevalence in these "successful" actualisations is comparable.

The only considerable difference seen in the model output compared to the experimental data is the broad proliferation of ST 257. In the experimental data shown in Figure 2, ST 257 quickly dies out, and as such there is little data available to infer its competitive strength in Figure 3. We theorise therefore that the model fits to allowing a ST with the least informing data to persist in order to act as a uniform competitive pressure against all STs, since a competition value of 0.5 is assumed against most other STs for which no information was available (the grey squares of Figure 3).
